# Clinical Evolution of Central Pontine Myelinolysis in a Patient with Alcohol Withdrawal: A Blurred Clinical Horizon

**DOI:** 10.1155/2016/6065259

**Published:** 2016-08-16

**Authors:** Abdul S. Mohammed, Prajwal Boddu, Dina F. Yazdani

**Affiliations:** ^1^Department of Internal Medicine, Advocate Illinois Masonic Medical Center, 2356 N. Elston Avenue, No. 306, Chicago, IL 60614, USA; ^2^Department of Internal Medicine, Advocate Illinois Masonic Medical Center, 856 W. Nelson Street, Apartment 2002, Chicago, IL 60657, USA; ^3^Deccan College of Medical Sciences, Kanchanbagh, Hyderabad, Telangana 500058, India

## Abstract

Central pontine myelinolysis (CPM), a potentially fatal and debilitating neurological condition, was first described in 1959 in a study on alcoholic and malnourished patients. It is a condition most frequently related to rapid correction of hyponatremia. Chronic alcoholism associated CPM tends to be benign with a favorable prognosis compared to CPM secondary to rapid correction of hyponatremia. We describe a normonatremic, alcoholic patient who presented with CPM after a rapid rise in his sodium levels. Our case illustrates the fact that CPM can manifest even in patients who are normonatremic at baseline. Rapid rises in sodium levels should be promptly reversed before clinical symptoms manifest in patient with risk factors for CPM irrespective of their baseline sodium levels. Furthermore, clinical evolution of CPM can be difficult to discern from the natural course of alcohol withdrawal delirium, requiring astuteness and maintenance of a high degree of clinical suspicion on the part of the physician.

## 1. Introduction

Central pontine myelinolysis (CPM), a potentially fatal and debilitating neurological condition, was first described in 1959 in a study on alcoholic and malnourished patients [1]. It is a condition most frequently related to rapid correction of hyponatremia. Involvement of the corticospinal tracts in the pons and midbrain, resulting in spastic quadriparesis and pseudobulbar palsy, is a characteristic neurological finding and renders this condition its name [2]. Progressive lethargy, quadriparesis, dysarthria, ophthalmoplegia, and ataxia are some of the frequent manifestations of this syndrome [3]. It was later discovered that the process of osmotic demyelination was not confined exclusively to the pons but also involved various extrapontine sites, a condition referred to as extrapontine myelinolysis (EPM) [4]. EPM generally occurs along with CPM but may occur in isolation as well [5]. Extrapyramidal features and myoclonus of EPM are some of the symptoms that represent potentially treatable manifestations of the disease and should be differentiated from CPM [6]. The term osmotic demyelination syndrome encompasses both the abovementioned entities. Comorbidities associated with higher incidence of CPM include dialysis, liver failure and transplantation, advanced lymphoma, carcinoma, cachexia, severe bacterial infections, acute hemorrhagic pancreatitis, chronic alcoholism, and pellagra [2, 7]. Chronic alcoholism associated CPM tends to be benign with a favorable prognosis compared to CPM secondary to rapid correction of hyponatremia [8, 9]. Also, CPM has been documented to occur in hypokalemic patients even with a steady rise in sodium levels [10, 11]. We describe a normonatremic, alcoholic patient who presented with CPM after a rapid rise in his sodium levels.

## 2. Case Report

A 57-year-old male with a known past medical history of squamous cell carcinoma of the oropharynx T2N0 on radiation therapy, chronic alcohol abuse, ulcerative colitis, and chronic obstructive pulmonary disease presented to the emergency department with 3 episodes of syncope and decreased oral intake over the week prior to presentation. He also reported difficulty swallowing solid foods over the past month. His last alcohol intake was 2 days prior to admission. On initial evaluation, vitals were significant for heart rate of 122 beats/min, blood pressure of 100/70 mmHg, respiratory rate of 20/min, and temperature of 37°C. Physical examination revealed a dry oral mucosa and decreased skin turgor. Laboratory studies were remarkable for sodium level of 126 mEq/L, potassium of 4.3 mEq/L, chloride of 86 mEq/L, bicarbonate of 28 mEq/L, BUN of 33 mg/dL, and creatinine of 4.2 mEq/L with a baseline of 0.6 mEq/L, platelets of 124,000/mm^3^, AST of 83 IU/L, ALT of 62 IU/L, ALP of 129 IU/L, and bilirubin of 1.9 mg/dL. Ammonia and albumin levels were noted to be within the normal range. Initial computerized tomography (CT) of the head was negative for acute intracranial process. The patient was started on IV fluids and admitted for further management.

**Figure 1:**
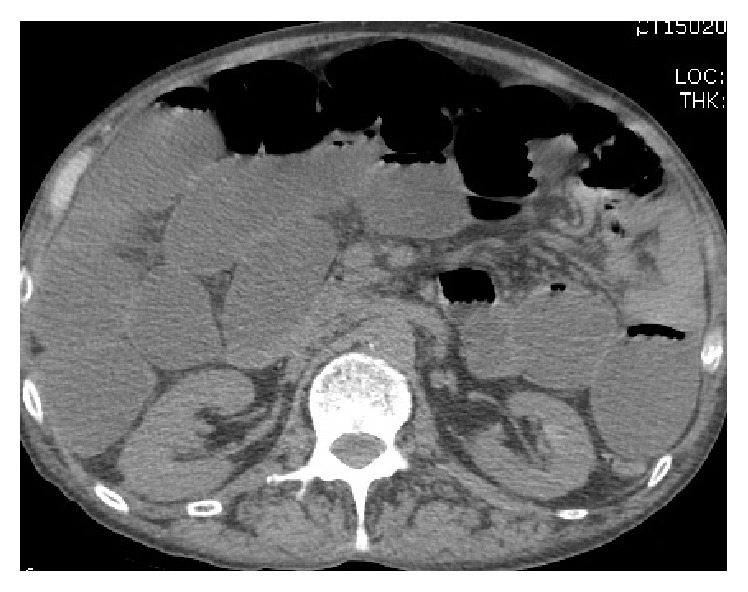
CT scan of the abdomen showing small bowel dilatation.

**Figure 2:**
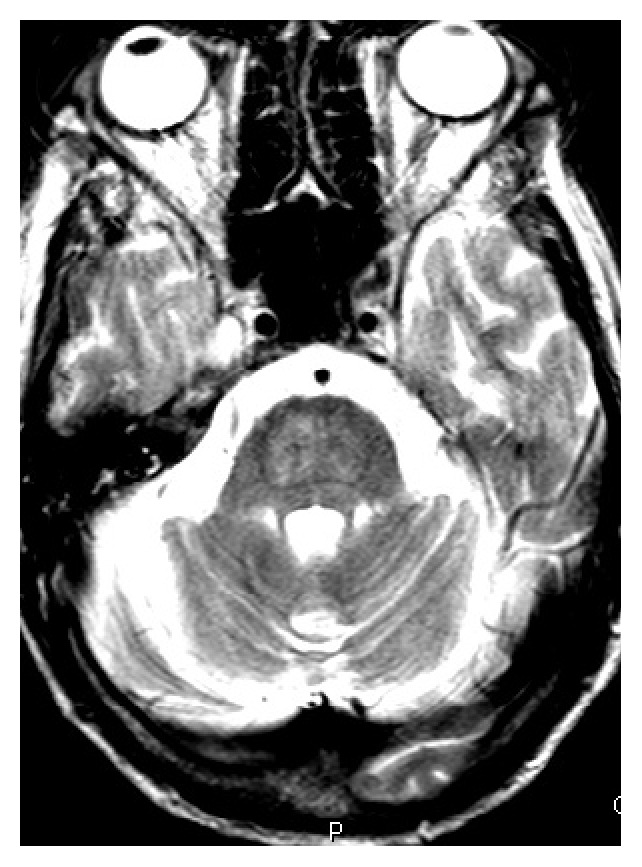
T2 magnetic resonance imaging demonstrating central pontine myelinolysis.

**Figure 3:**
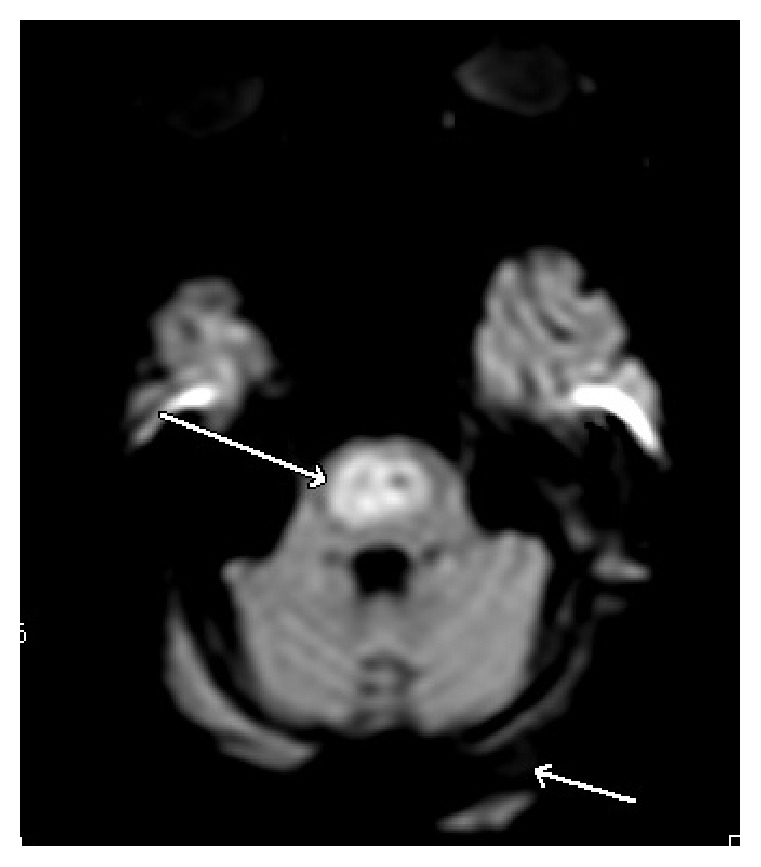
Diffusion weighted MRI image of our patient demonstrating central pontine myelinolysis.

Over the next few days, the patient's hydration status improved and his appetite increased. His creatinine trended down and his sodium level increased to 130 mEq/L. On fourth day of admission, the patient was noted to be combative towards the medical staff. He also started showing signs of alcohol withdrawal with tachycardia, diaphoresis, tremors, anxiety, and confusion. Examination revealed progressively worsening abdominal distention. Sodium levels rose steadily to 140 mEq/L over the next four days. On the eighth day of admission, intravenous (IV) fluids were changed to D5W in light of the increasing sodium level. A CT scan of the abdomen was obtained for the increasing abdominal distention, which revealed dilatation of the small bowel (see Figure 1) extending from the proximal jejunum to the distal ileum as well as marked dilatation of the cecum extending to the proximal descending colon, suggestive of adynamic ileus. Cirrhosis of the liver and recto sigmoid diverticula were also noted. A nasogastric (NG) tube was placed and put to wall suction. Flexible sigmoidoscopy was performed but the scope had to be withdrawn prematurely due to the risk of perforation of diverticuli. He continued to be agitated and confused, requiring frequent doses of lorazepam to control his symptoms. On days 9 and 10, patient's sodium level increased steeply from 142 mEq/L to 151 and 159 mEq/L, respectively. On day 12, the patient had decreased response to verbal and physical stimuli. It was noted at this time that the patient was no longer on lorazepam. Ongoing free water losses from diarrhea, NG suction, and extensive third spacing into the bowel delayed the rapid reversal of hypernatremia despite aggressive IV fluid hydration. He became increasingly lethargic and developed worsening dysarthria and dysphagia. Neurological exam revealed disconjugate gaze with exotropia, flaccid quadriparesis, and absent deep tendon reflexes. Magnetic resonance imaging (MRI) of the brain (see Figures 2 and 3) demonstrated increased T2 and flair signals in the central basis pontis consistent with central pontine myelinolysis.

**Figure 4:**
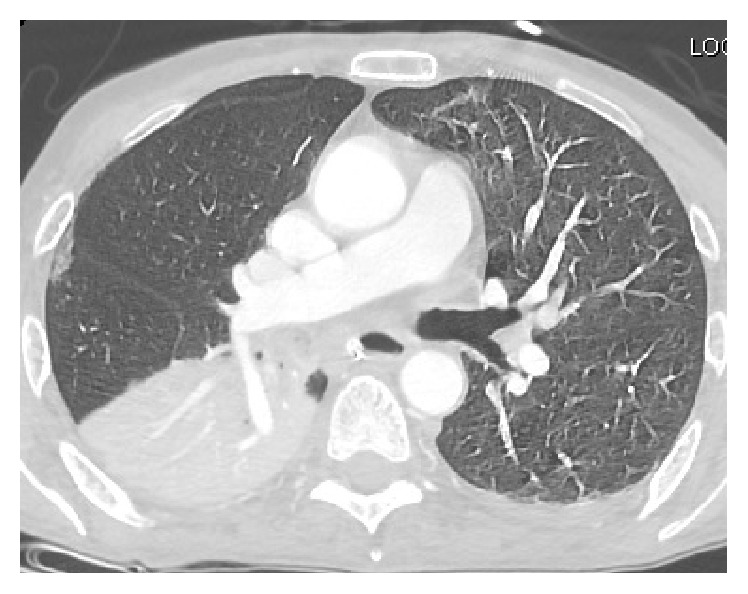
CT scan of the chest showing aspiration pneumonitis.

On the 13th day of the hospital course, the patient started developing respiratory distress with tachypnea into the 30 s; examination revealed rapid shallow breathing and use of accessory muscles. CT scan of the chest showed significant debris in the right main stem bronchus along with collapse of the right lower lobe consistent with aspiration pneumonitis (see Figure 4). Per patient's family's wishes, comfort measures were initiated and patient passed away later that day.

## 3. Discussion

CPM usually occurs with rapid correction of hyponatremia but may also occur in normonatremic and hypernatremic individuals [12–14]. CPM has also been described in patients with acute hypernatremia resulting from diabetes insipidus [15, 16]. Rarely, a CPM like condition developing after acute correction of hypernatremia has been described. The patient discussed in this report showed a dramatic recovery in his clinical and radiological manifestations of disease suggesting that this syndrome is probably operated by a different pathogenic mechanism [17]. The association between CPM and rapid correction of hyponatremia was first suggested by Norenberg et al. in a cohort of 12 CPM patients, the majority of whom had rapid and sustained correction of sodium levels into the hypernatremic range [18]. A subsequent study, by Ayus et al., identified rapid correction of sodium into the hypernatremic range especially in the setting of hepatic encephalopathy to be a contributing factor in the demyelinating process [19]. It is believed that rapid corrections of sodium levels create hyperosmotic stresses at regions of compact grey-white matter interdigitations leading to cellular swelling and compression induced myelinolysis of fiber tracts. Other hypotheses suggest that rapid correction may lead to endothelial injury created gaps in blood brain barrier resulting in release of unidentified myelinotoxic blood derived factors [20].

The neuropsychiatric symptoms of CPM can be similar to those seen in alcohol withdrawal. Furthermore, the ascription of subtle neurological changes to the development of CPM in a patient experiencing alcohol withdrawal can be difficult due to the confounding effects of sedative use. Alcohol delirium tremens typically lasts 1–5 days from the onset of its presentation. A prolonged encephalopathy beyond the expected duration of delirium tremens should raise concern for complicating neuropathologies and prompt further workup [21]. CPM typically presents 2–6 days after an acute rise in sodium level. Our patient continued to have neuropsychiatric symptoms for more than a week from the onset of delirium. There was a rapid rise in sodium between days 9 and 10. It was on day 12, when the patient continued to be obtunded while not being on sedatives, that the diagnosis of CPM was first entertained. Our patient did not present with active visual hallucinosis or agitated delirium to suggest alcoholic psychosis. Secondly, the CPM had to be distinguished from Wernicke encephalopathy, a condition that may occur along with it as an infrequently recognized combination [22, 23]. Our patient had eye movement abnormalities, a finding that was noted in 25% of patients with CPM in one study [24]. Our patient was treated adequately with intravenous thiamine for the duration of the hospital stay. Oculomotor dysfunction in spite of adequate thiamine administration pointed against the diagnosis of Wernicke encephalopathy [22].

Magnetic resonance imaging is the imaging sequence of choice in detecting CPM lesions. Areas affected by myelinolysis are hypoattenuating and typically involve the basis pontis with sparing of pontine tegmentum. Basal ganglia and thalamus may also be involved, indicating extrapontine myelinolysis. Lesions are typically tridentate and tend to be hyperintense on T2 and hypointense on T1 without enhancement even after administration of contrast [25, 26]. CT and MRI findings may lag behind the clinical manifestations by up to two weeks. However, diffusion weighted imaging (DWI) may be able to detect lesions as early as 24 hours from the manifestation of clinical symptoms [27].

Given the potentially severe and permanent adverse consequences of CPM, prevention is essential. The ideal management hinges upon identifying patients whose sodium levels have been corrected rapidly as high risk and pursuing rescue strategies, including the use of DDAVP to facilitate reversal of acute rise in sodium levels [28]. Prompt recognition and rapid resolution of treatment-induced hypernatremia are imperative in reversing the processes underlying demyelination before clinical symptoms manifest. Our patient had all the major risk factors of CPM including alcoholism, malnutrition, and hypokalemia [13]. Although our patient's hypernatremia was recognized promptly, he continued to have significant free water losses from large volume diarrhea, nasogastric suction, and third spacing, preventing the rapid reversal of sodium levels. Continued free water losses are the most frequent reason for miscalculation of free water deficits and appropriate correction in sodium levels. The combination of acute hypernatremia, hypokalemia, and chronic alcoholism had precipitated CPM in our patient. Although acute hypernatremia and its slow reversal may have contributed significantly to the manifestation of the disease, it can only be speculated if our patient would have developed CPM from the effects of alcoholism, malnutrition, and hypokalemia alone without the additional contribution of hypernatremia.

## 4. Conclusion

Our case illustrates the fact that CPM can manifest even in patients who are normonatremic at baseline. Rapid rises in sodium levels should be promptly reversed before clinical symptoms manifest in patients with risk factors for CPM irrespective of their baseline sodium levels. Furthermore, clinical evolution of CPM can be difficult to discern from the natural course of alcohol withdrawal delirium, requiring astuteness and maintenance of a high degree of clinical suspicion on the part of the physician.
